# Long‐Term Risk of Type 2 Diabetes Associated With Topical Corticosteroid Use: A Nationwide Population‐Based Cohort Study in Korea

**DOI:** 10.1155/adpp/8843438

**Published:** 2026-05-28

**Authors:** Seungwoo Kim, Ji Hui An, In Sun Ryou, Yongho Jee

**Affiliations:** ^1^ FELZ Inc., Goyang-si, Gyeonggi-do, South Korea; ^2^ International Health Promotion Center, Ewha Womans University Seoul Hospital, Seoul, South Korea; ^3^ Department of Family Medicine, Ewha Womans University Seoul Hospital, Seoul, South Korea; ^4^ Advanced Biomedical Research Institute, Ewha Womans University Seoul Hospital, Seoul, South Korea

**Keywords:** diabetes mellitus, proportional hazards models, steroids, survival analysis

## Abstract

Steroid‐induced diabetes mellitus (SIDM) is a well‐known side effect of corticosteroid agents. While diabetes induced by systemic corticosteroids has been extensively studied for over 60 years, evidence regarding the association between topical corticosteroids and Type 2 diabetes remains limited. This study aimed to investigate the relationship between topical corticosteroid use and the risk of Type 2 diabetes. A nationwide, population‐based cohort study was conducted using data from the Korea National Health Insurance Service National Sample Cohort Database (NHIS‐NSC2). A total of 186,057 adults without prior Type 2 diabetes were included in the cohort for follow‐up. Among these, 14,944 adults who had been treated with topical corticosteroids and 171,113 adults with no history of topical corticosteroid use were included and followed prospectively for approximately 9 years. The primary outcome was the incidence of Type 2 diabetes following topical corticosteroid use, with additional analyses by frequency and potency of corticosteroid use. Kaplan–Meier curves and Cox proportional hazard models were used to estimate hazard ratios (HR) for Type 2 diabetes. The use of topical corticosteroids was significantly associated with an increased risk of Type 2 diabetes (adjusted HR [aHR] 1.13, 95% CI 1.06–1.20). The high‐frequency corticosteroid group demonstrated the highest risk (aHR 1.27, 95% CI 1.09–1.48), while the moderate‐potency corticosteroid group also showed a significant increase in risk (aHR 1.15, 95% CI 1.04–1.27). Notably, the diabetes risk in the moderate‐potency group was comparable to that observed in the systemic corticosteroid group (aHR 1.20, 95% CI 1.14–1.26). In this study, the use of topical corticosteroids was significantly associated with the incidence of Type 2 diabetes. These findings highlight the importance of considering diabetes risk factors when prescribing topical corticosteroids.

## 1. Introduction

In Korea, the prevalence of diabetes has shown an increasing trend, rising from 12.4% in 2011 to 13.0% in 2014 and 14.4% in 2016. According to the Korean Ministry of Health and Welfare, the medical expenses for diabetes in Korea have sharply increased from 1.4 trillion won in 2010 to 2.2 trillion won in 2017, which not only increases the medical burden on individuals and society but also hinders the stabilization of the public healthcare system [1].

Diabetes is a representative metabolic disorder characterized by hyperglycemia due to defects in insulin secretion or action, and it is more frequently associated with chronic diseases compared to the general population without diabetes. According to Kim et al., 88% of patients with diabetes in Korea had at least one comorbid condition such as hypertension or hyperlipidemia, which are well‐known risk factors for cardiovascular diseases [2]. In 2018, diabetes ranked as the sixth leading cause of death in Korea, following cancer, heart disease, pneumonia, cerebrovascular disease, and suicide [1].

Corticosteroids are widely used for their anti‐inflammatory and immunosuppressive effects in treating numerous conditions, ranging from chronic diseases such as chronic pulmonary disease, rheumatoid disorders, and psoriasis to acute conditions like gout, various malignancies, and organ transplants [3]. However, due to their various side effects, caution is required in their use. Among these side effects, steroid‐induced diabetes mellitus (SIDM) is one of the adverse effects reported 60 years ago, where steroids absorbed through medication interfere with insulin action or promote glucose synthesis in the liver, leading to increased blood glucose levels and elevating the risk of Type 2 diabetes [4].

Topical corticosteroids, which are applied to the skin, are commonly used in dermatology for a wide range of indications, from chronic inflammatory skin diseases such as psoriasis and atopic dermatitis to itching skin conditions like eczema. They are developed in various strengths and forms to be easily used by infants and the elderly [5]. Topical corticosteroids may be perceived as safer than oral medications due to their application on the skin. However, recent studies have shown that because their particles are small, they can affect the entire body through the bloodstream even when applied to the skin, and they are dose‐dependent just like oral medications [6]. However, there is significantly less evidence compared to oral medications, and the existing studies were conducted in Western countries, which may differ from the general characteristics of the Korean population, pointing to limitations in applying these findings to Korean clinical practice. Additionally, no cohort studies have used family history of diabetes and body mass index (BMI), which are risk factors for SIDM, as adjustment variables, leading to interpretative limitations.

Therefore, this study aims to investigate the risk of Type 2 diabetes onset associated with the use of topical corticosteroids in the Korean population, using variables such as family history of diabetes and BMI, which were identified as limitations in previous studies. The study will differentiate the risk according to the use, frequency of use, and potency of topical corticosteroids.

## 2. Materials and Methods

### 2.1. Data Collection and Selection of Study Participants

This study utilized the National Health Insurance Service National Sample Cohort 2.0 (NHIS‐NSC2) provided by the NHIS. The NSC2 is a research database established in June 2017, containing information from individuals’ NHIS enrollment, including hospital and clinic utilization data, as well as health examination results. As of 2006, it includes a cohort of 1 million people (approximately 2% of the entire population) representative of the national population, encompassing 14 years of information from 2002 to 2015.

Our study population consisted of subjects aged 19 years or older with a claims history between January 1, 2006, and December 31, 2007, from the NHIS‐NSC2. The topical corticosteroid use group was defined as individuals who were prescribed topical corticosteroids two or more times between 2006 and 2007. The period from January 1, 2002, to December 31, 2005, was set as a wash‐out period, during which participants who could influence the study were excluded. First, individuals with primary or secondary outpatient diagnoses corresponding to E.11.X, those who had been prescribed hypoglycemic agents, had fasting blood glucose levels of 126 mg/dL or higher, or who self‐reported having diabetes during medical examinations were defined as Type 2 diabetes patients and excluded from the study. Furthermore, to eliminate the effect of topical corticosteroid use, participants with a prescription for topical corticosteroids during the wash‐out period were excluded, as were those without health examination data.

### 2.2. Definition and Classification of Variables

The variables used in the study were selected and defined as shown in Supporting Table 1, based on the factors influencing corticosteroid use identified in previous studies through a literature review. According to Suh and Park, age, BMI, family history of diabetes, history of impaired glucose tolerance, dosage, and duration of treatment are known risk factors for diabetes related to steroid use [7]. In addition, hypertension, smoking, alcohol consumption, and fasting blood glucose, which are known risk factors for Type 2 diabetes, were also selected as variables. More recently, it has been confirmed that the incidence of diabetes is higher in patients with psoriasis compared to the general population, and this was used as a confounding variable [8].

The independent variable, topical corticosteroids, the confounding variable, and systemic corticosteroids, were classified using the active ingredient codes employed by the Sample Cohort 2.0, derived from the entire range of corticosteroid types developed as medications. As shown in Table 1, the active ingredient codes consist of a total of nine components, with the 7th indicating the route of administration and the 8th and 9th representing the formulation. According to the operational definitions in Table 2, corticosteroids designed in formulations such as creams and lotions, intended to act directly on the body surface like the skin, were classified as topical corticosteroids, while eye ointments were excluded from this classification. Systemic corticosteroids were defined as formulations acting on the entire body, such as oral medications or injections. The active ingredient codes were based on the status as of March 2008, and the types of drugs used in the analysis are listed in supporting Table 2. Topical corticosteroids can be classified into seven potency levels based on the degree of vasoconstriction, with higher potency indicating a stronger drug effect [9]. In addition, the frequency of topical corticosteroid use was categorized based on the number of prescriptions during the 2‐year baseline period (2006–2007). Specifically, four groups were defined: 0 (nonusers), 1 time, 2–4 times, and 5 or more times. This classification was designed to evaluate the dose‐response relationship based on prescription frequency.

**TABLE 1:** Characteristics according to the use of topical corticosteroids (*n* = 186,057).

	**Nonuse *n* = 171,113 (92.0%)**	**Use *n* = 14,944 (8.0%)**

Sex		
Male	94,898 (55.5^∗^)	6508 (43.6^∗^)
Female	76,215 (44.5^∗^)	8436 (56.5^∗^)
Age (years)		
Mean (SD)	42.2 (14.0^∗^)	44.6 (14.5)^∗^
< 30	37,988 (22.2)	2831 (18.9)
30–39	42,100 (24.6)	3073 (20.6)
40–49	43,366 (25.3)	3827 (25.6)
50–59	26,133 (15.3)	2719 (18.2)
60–69	14,493 (8.5)	1675 (11.2)
70	7033 (4.1)	819 (5.5)
BMI (kg/m^2^)		
Mean (SD)	23.5 (3.3)	23.5 (3.2)
< 18.5	8269 (4.8)	749 (5.0)
18.5–25	110,233 (64.4)	9529 (63.8)
25–30	46,871 (27.4)	4185 (28.0)
> 30	5713 (3.3)	480 (3.2)
Smoking		
Ever‐smoker	70,551 (41.6^∗^)	4861 (32.9^∗^)
Nonsmoker	98,855 (58.4^∗^)	9926 (67.1^∗^)
Alcohol		
More than 1∼2 times/week	46,859 (27.7^∗^)	3461 (23.4^∗^)
Less than 2∼3 times/month	122,485 (72.3^∗^)	11,321 (76.6^∗^)
FBS (mg/dL) (SD)	93.6 (16.8)	93.3 (15.7)
< 100	127,733 (74.6)	11,227 (75.1)
100–125	38,952 (22.8)	3358 (22.5)
Psoriasis	536 (0.3^∗^)	495 (3.3^∗^)
Hypertension	16,636 (9.7^∗^)	1821 (12.2^∗^)
Hyperlipidemia	20,994 (12.3^∗^ ^)^	2024 (13.5^∗^)
DM family history	28,864 (16.9^∗^)	2367 (15.8^∗^)
Systemic CS	22,164 (13.0^∗^)	4563 (30.5^∗^)
Income status		
Lowest	27,746 (16.6)	2535 (17.5)
Below average	29,326 (17.6)	2427 (16.8)
Average	32,711 (19.6)	2641 (18.2)
Above average	38,612 (23.1)	3344 (23.1)
Highest	38,534 (23.1)	3542 (24.4)
T2D incidence during F/U	12,318 (7.2)^∗^	1361 (9.1)^∗^

*Note:* BMI, body mass index (kg/m^2^); FBS, fasting blood sugar (mg/dL); CS, corticosteroid.

Abbreviations: F/U, follow‐up; SD, standard deviation; T2D, Type 2 diabetes.

^∗^Statistically significant difference between the topical corticosteroid use and nonuse groups were assessed using chi‐square tests for categorical variables and *t*‐tests for continuous variables (*p* < 0.05).

**TABLE 2:** Association between topical corticosteroid use and risk of Type 2 diabetes.

Predictor	Crude	Model 1	Model 2
	HR (95% CI)	HR (95% CI)	HR (95% CI)
TCS use	1.29 (1.22–1.36)	1.13 (1.07–1.20)	1.13 (1.06–1.20)
Age			
< 30		Ref.	Ref.
30–39		1.84 (1.65–2.05)	1.84 (1.65–2.05)
40–49		3.99 (3.61–4.40)	3.99 (3.61–4.40)
50–59		7.01 (6.36–7.74)	7.01 (6.36–7.74)
60–69		10.10 (9.12–11.18)	10.10 (9.12–11.18)
≥ 70		11.50 (10.28–12.85)	11.50 (10.28–12.85)
Sex (Model 1)		1.09 (1.03–1.15)	1.09 (1.03–1.15)
BMI (Model 1)		1.11 (1.10–1.11)	1.11 (1.10–1.11)
Smoking (Model 2)			1.21 (1.15–1.28)
Alcohol (Model 2)			0.91 (0.86–0.95)
Psoriasis (Model 2)			0.90 (0.70–1.14)
Hypertension (Model 2)			1.74 (1.67–1.82)
Hyperlipidemia (Model 2)			1.27 (1.21–1.33)
DM family history (Model 2)			1.32 (1.26–1.38)
FBS (mg/dL) (Model 2)			
< 100			Ref.
100–125			2.18 (2.10–2.27)
Systemic CS (Model 2)			1.20 (1.14–1.26)
Income status (Model 2)			
Lowest			1.01 (0.95–1.07)
Below average			1.03 (0.97–1.10)
Average			Ref.
Above average			1.00 (0.94–1.06)
Highest			0.88 (0.83–0.93)

*Note:* BMI, body mass index (kg/m^2^); FBS, fasting blood sugar (mg/dL); CS, corticosteroid.

Abbreviation: DM, diabetes mellitus.

Potency was determined according to vasoconstrictor assay–based levels and mapped to the ATC classification system. The original seven levels were regrouped into three broader categories (mild, moderate, and high potency), as summarized in Supporting Table 3. Accordingly, to examine the effect of potency, this study reclassified the seven potency levels into high, medium, and low potency groups and conducted a stratified analysis. The classification criteria are shown in supporting Table 3. The occurrence of Type 2 diabetes, the dependent variable, was determined based on either of the following criteria: (1) if the KCD code E11.X, classified as Type 2 diabetes, appeared three or more times as a primary or secondary diagnosis during the follow‐up period, the first occurrence was considered the onset or (2) if the individual was prescribed a hypoglycemic agent.

### 2.3. Statistical Analysis

The analysis was conducted using Kaplan–Meier curves to perform stratified analyses based on the use and nonuse groups, drug potency, and usage frequency (0, 1, 2–4, 5 or more times/2 years). Statistical significance was confirmed using the log‐rank test. The start of patient follow‐up was defined as the point of the second prescription of topical corticosteroids, and patients who did not develop diabetes during the follow‐up period or those who died were censored. The Cox proportional hazards model was applied after reviewing the Schoenfeld residual test assumptions, and all models satisfied the proportionality assumption. Covariates were selected based on their clinical relevance and prior literature. These included demographic characteristics (age and sex), metabolic factors (BMI, blood pressure, and fasting glucose), lifestyle behaviors (smoking and alcohol), and comorbidities (psoriasis, hypertension, hyperlipidemia, and family history of diabetes). This multivariable adjustment was intended to reduce potential confounding and isolate the independent association between topical corticosteroid use and diabetes risk. For each group, crude and adjusted hazard ratios (aHR) were calculated to identify risk factors. Statistical analysis was performed using SAS version 9.4, and a significance level of 5% was set for all analyses. Supporting Information: Detailed variable definitions, drug classifications, and additional subgroup analyses are provided in Supporting Tables 1–5.

## 3. Results

**FIGURE 1:**
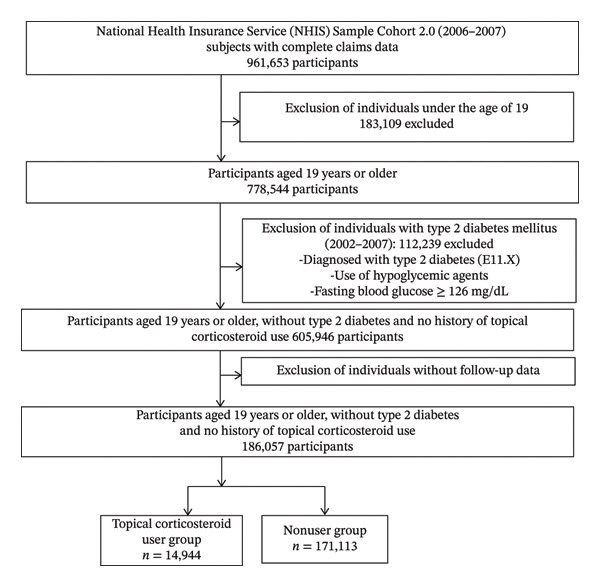
Flowchart of study population.

A total of 961,653 individuals with claim records from 2006 to 2007 in the NHIS‐NSC2 were initially identified, of whom 778,544 were aged ≥ 19 years. After excluding individuals with a diagnosis of Type 2 diabetes during the washout period (*n* = 112,239) and those previously prescribed topical corticosteroids (*n* = 60,359), 186,057 participants with health examination data were included in the final cohort and followed until 2015 (Figure 1). Among them, 14,944 individuals (8.0%) received two or more prescriptions for topical corticosteroids and were classified as the user group; the remaining 171,113 individuals (92.0%) formed the nonuser group.

Baseline characteristics differed significantly between the two groups (Table 3). The user group had a higher mean age (44.6 vs. 42.2 years, *p* < 0.05), a higher proportion of females (56.5% vs. 44.5%, *p* < 0.05), greater prevalence of psoriasis (3.3% vs. 0.3%, *p* < 0.05), hypertension (12.2% vs. 9.7%), and systemic corticosteroid use (30.5% vs. 13.0%). Meanwhile, smoking and family history of diabetes were more prevalent in the nonuser group. During a mean follow‐up of 8.5 years, the incidence of Type 2 diabetes was 9.1% in the user group and 7.2% in the nonuser group. This corresponds to an absolute risk difference of 1.9%, which supports the findings from the aHR. Subgroup characteristics by frequency and potency of topical corticosteroid use are presented in Supporting Tables 4 and 5. A dose‐response trend was observed, with higher frequency and moderate potency associated with elevated diabetes risk.

**TABLE 3:** Associations between frequency of topical corticosteroid (TCS) use and risk of Type 2 diabetes.

Predictor	Crude	Model 1	Model 2
	HR (95% CI)	HR (95% CI)	HR (95% CI)
TCS frequency			
0	ref.	ref.	ref.
1	1.09 (1.04–1.15)	1.07 (1.02–1.25)	1.07 (1.02–1.13)
2–4	1.24 (1.17–1.32)	1.11 (1.05–1.18)	1.12 (1.05–1.20)
≥ 5	1.79 (1.57–2.05)	1.35 (1.19–1.55)	1.27 (1.09–1.48)
Age (Model 1)			
< 30		ref.	ref.
30–39		1.84 (1.65–2.05)	1.84 (1.65–2.05)
40–49		3.99 (3.61–4.40)	3.99 (3.61–4.40)
50–59		7.01 (6.36–7.74)	7.01 (6.36–7.74)
60–69		10.09 (9.11–11.17)	10.09 (9.11–11.17)
≥ 70		11.49 (10.28–12.85)	11.49 (10.28–12.85)
Sex (Model 1)		1.09 (1.03–1.14)	1.09 (1.03–1.14)
BMI (Model 1)		1.11 (1.10–1.11)	1.11 (1.10–1.11)
Smoking (Model 2)			1.21 (1.15–1.28)
Alcohol (Model 2)			0.91 (0.86–0.95)
Psoriasis (Model 2)			0.87 (0.68–1.11)
Hypertension (Model 2)			1.74 (1.67–1.82)
Hyperlipidemia (Model 2)			1.27 (1.21–1.33)
DM family history			1.32 (1.26–1.38)
FBS (mg/dL) (Model 2)			
< 100			
100–125			2.19 (2.10–2.27)
Systemic CS (Model 2)			1.20 (1.14–1.26)
Income status (Model 2)			
Lowest			1.01 (0.95–1.07)
Below average			1.03 (0.97–1.10)
Average			ref
Above average			1.00 (0.94–1.06)
Highest			0.88 (0.83–0.93)

*Note:* BMI, body mass index (kg/m^2^); TCS, topical corticosteroids.

Abbreviations: aHR, adjusted hazard ratio; CI, confidence interval; DM, diabetes mellitus; FBG, fasting blood glucose; HR, hazard ratio.

**FIGURE 2:**
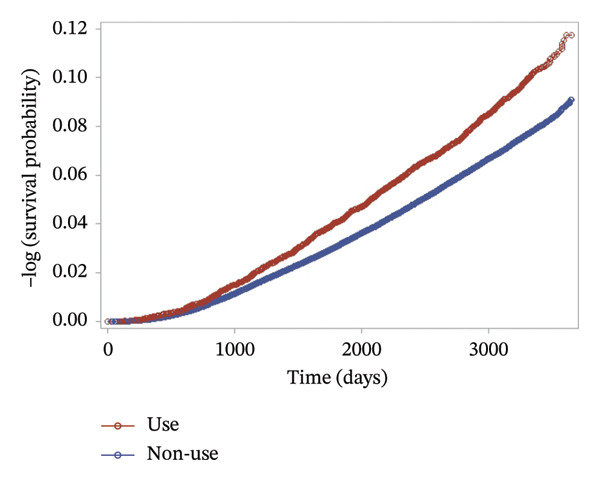
Kaplan–Meier survival curve according to the use of topical corticosteroids (log‐rank test *p* < 0.001).

Kaplan–Meier survival analysis revealed a significant difference in the cumulative incidence of Type 2 diabetes between the topical corticosteroid user and nonuser groups (Figure 2). The separation between the two curves became apparent approximately 2 years after baseline and widened throughout the follow‐up period (log‐rank *p* < 0.001).

**FIGURE 3:**
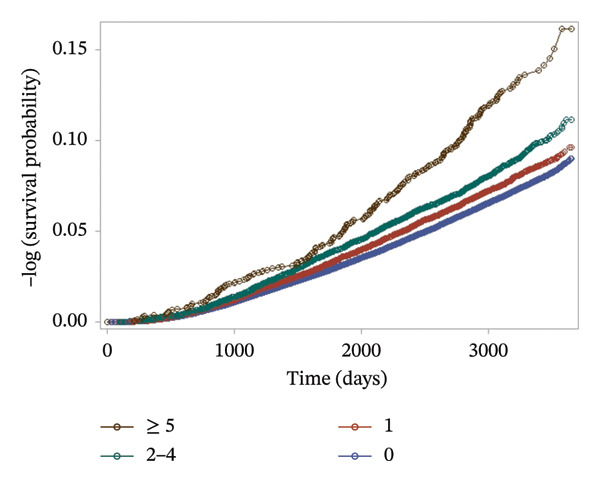
Kaplan–Meier survival curve according to the frequency of topical corticosteroids (log‐rank test *p* < 0.001).

When stratified by the frequency of topical corticosteroid use (0, 1, 2–4, and ≥ 5 times within 2 years), a clear dose‐response pattern was observed (Figure 3). The cumulative incidence of Type 2 diabetes was highest in the ≥ 5‐use group, followed by the 2–4‐use, 1‐use, and nonuse groups. Although exact cumulative proportions are not labeled within the figure itself, incidence rates such as 7.1% in the nonuser group and 12.1% in the highest‐frequency group were derived from Supporting Table 4.

**FIGURE 4:**
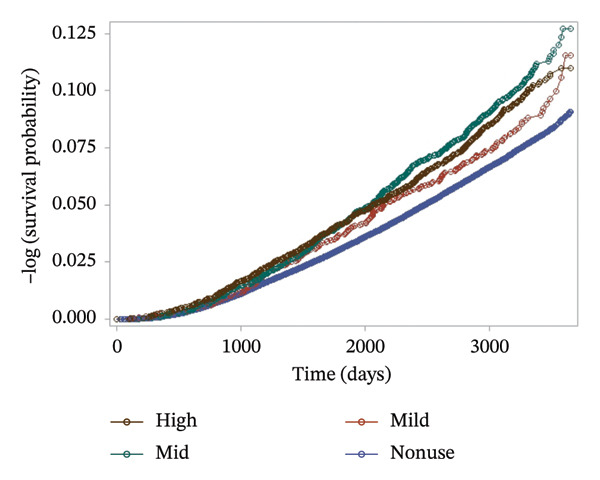
Kaplan–Meier survival curve according to the potency of topical corticosteroids (log‐rank test *p* < 0.001).

A similar trend was observed when participants were grouped by the potency of prescribed topical corticosteroids (mild, mid, and high potency). The mid‐potency group exhibited the highest cumulative incidence of diabetes, followed by the high‐ and mild‐potency groups, with all user groups showing significantly elevated risk compared to nonusers (Figure 4; log‐rank *p* < 0.001). These findings are consistent with incidence rates provided in Supporting Table 5.

**FIGURE 5:**
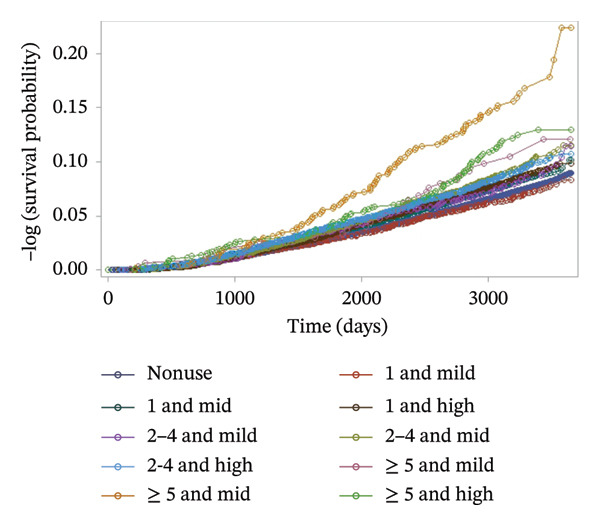
Kaplan–Meier survival curve according to the frequency and potency of topical corticosteroids (log‐rank test *p* < 0.001).

Finally, Figure 5 presents the combined effect of frequency and potency on diabetes risk. Participants in the high‐frequency (≥ 5 uses) and mid‐potency subgroup demonstrated the highest cumulative incidence over time, suggesting a synergistic risk effect. In contrast, participants with mild potency and low frequency (1 use) had a risk comparable to the nonuser group. While the figures visualize overall trends, quantitative incidence rates supporting these observations are detailed in Supporting Tables 4 and 5. In Cox proportional hazards models (Table 1), topical corticosteroid use was associated with a significantly increased risk of developing Type 2 diabetes (crude HR 1.29, 95% CI: 1.22–1.36). This association persisted after adjustment for age, sex, BMI (Model 1: aHR 1.13, 95% CI: 1.07–1.20) and additional covariates including smoking, alcohol use, psoriasis, systemic corticosteroid use, family history of diabetes, hypertension, hyperlipidemia, fasting blood glucose, and income (Model 2: aHR 1.13, 95% CI: 1.06–1.20).

Stratified analysis by prescription frequency (Table 2) further demonstrated a dose‐response relationship: compared to nonusers, the adjusted HRs for developing Type 2 diabetes were 1.07 (95% CI: 1.02–1.13) for 1 use, 1.12 (95% CI: 1.05–1.20) for 2–4 uses, and 1.27 (95% CI: 1.09–1.48) for ≥ 5 uses, after full covariate adjustment.

Stratified analysis by prescription frequency (Table 2) further demonstrated a dose‐response relationship: compared to nonusers, the aHRs for developing Type 2 diabetes were 1.07 (95% CI: 1.02–1.13) for 1 use, 1.12 (95% CI: 1.05–1.20) for 2–4 uses, and 1.27 (95% CI: 1.09–1.48) for ≥ 5 uses, after full covariate adjustment.

## 4. Discussion

This study investigated the association between the use of topical corticosteroids and the risk of developing Type 2 diabetes among Koreans, using data from the NHIS‐NSC2. The findings revealed that individuals using topical corticosteroids exhibited a higher risk of developing Type 2 diabetes compared to nonusers (aHR 1.13, 95% CI 1.06–1.20). Furthermore, a dose‐response relationship was observed, with the risk increasing as the frequency of corticosteroid use escalated (1 use, aHR 1.07, 95% CI 1.02–1.13; 2–4 uses, aHR 1.12, 95% CI 1.05–1.20; 5 or more uses, aHR 1.27, 95% CI 1.09–1.48), reaching a risk comparable to that of systemic corticosteroid use (aHR 1.20, 95% CI 1.14–1.26).

Several studies have suggested an association between topical steroid use and the risk of new‐onset DM. An analysis of the PHARMO Record Linkage System in the Netherlands found a significant dose‐dependent relationship between topical corticosteroid use and diabetes risk (OR 1.20, 95% CI 1.06–1.35), although no correlation with corticosteroid potency was observed [10]. Similarly, case‐control studies in Denmark (OR 1.25, 95% CI 1.23–1.28) and the United Kingdom (OR 1.27, 95% CI 1.23–1.31) demonstrated an association between topical corticosteroid use and diabetes. However, only the Danish study revealed a dose‐dependent effect. A Danish cohort study further supported these findings, reporting an increased risk of incident Type 2 diabetes with a dose‐dependent aHR (HR 1.27, 95% CI 1.26–1.29) [6]. Additionally, a meta‐analysis of four case‐control studies, involving over 3500 participants, confirmed a dose‐dependent relationship between topical steroid use and diabetes risk, regardless of corticosteroid potency (OR 1.24, 95% CI 1.15–1.34) [11]. These findings underscore the association between topical corticosteroid use and the increased incidence of diabetes.

Steroids contribute to hyperglycemia and increase the risk of diabetes through several well‐established mechanisms. They influence genes involved in carbohydrate metabolism, such as phosphoenolpyruvate carboxykinase (PEPCK), by upregulating their transcription. This process enhances hepatic gluconeogenesis, leading to elevated endogenous glucose production [11–14]. Additionally, steroids impair insulin sensitivity by reducing GLUT‐4 translocation in skeletal muscle and adipose tissue, thereby decreasing peripheral glucose uptake. Steroids are also known to promote muscle protein breakdown and lipolysis in adipose tissue, resulting in unfavorable changes in body composition and increased systemic insulin resistance [11, 13, 15, 16]. Chronic glucocorticoid exposure further exerts direct cytotoxic effects on pancreatic β‐cells, impairing insulin production and secretion in a dose‐ and duration‐dependent manner [11, 17–19].

These effects may not be limited to systemic administration. Topical steroids, despite their localized application, can exert systemic effects due to percutaneous absorption. Percutaneous absorption involves the passage of the drug through the epidermis and dermis into systemic circulation [20]. Although less than 2% of a single topical steroid application (e.g., hydrocortisone) is absorbed after remaining on the skin for more than a day [21], several factors influence the degree of absorption. These factors include the amount of steroid applied, the frequency of application, the surface area treated, the potency of the steroid, the use of occlusion, among other factors [20]. Skin conditions that disrupt the barrier function, such as eczema, can further enhance absorption, increasing the likelihood of systemic effects [22]. The stratum corneum serves as the primary barrier to percutaneous absorption [23], but its thickness and integrity vary by body site and individual factors. Moreover, the stratum corneum acts as a reservoir, allowing sustained release and absorption of the steroid for up to 5 days after a single application [24]. These systemic exposures, although minimal, can mimic the metabolic consequences of systemic steroid use, such as glucose dysregulation and increased diabetes risk. In our study, the frequency and amount of topical steroid use were significantly associated with an increased risk of diabetes. This observation can be explained by the mechanisms of percutaneous absorption.

However, we also found that the risk of diabetes did not increase significantly with the potency of topical steroids. High‐potency groups exhibited a similar or even lower risk compared to medium‐potency groups. According to Li et al., two studies confirmed that adherence to medication among patients with atopic dermatitis who had a phobia of topical corticosteroid use was significantly lower than in those without such fears (49.4% vs 14.1%, 29.3% vs 9.8%) [25]. This reduced adherence may be attributed to past experiences of side effects, such as skin thinning, concerns about systemic effects, or fears of reduced efficacy with long‐term use [25–27]. Furthermore, patients are often cautioned against using high‐potency corticosteroids for more than 3 weeks or applying them to sensitive areas like the face, which may reinforce these concerns through educational efforts. As a result, even when high‐potency corticosteroids are prescribed at the same frequency as lower‐potency medications, actual usage may be reduced due to concerns about duration of use and potential side effects [9]. Additionally, high‐potency corticosteroids are more commonly prescribed for patients with mild psoriasis, whereas medium‐potency corticosteroids are frequently used for patients with atopic dermatitis. Since the affected area is generally larger in patients with atopic dermatitis compared to those with mild psoriasis, differences in application area may have influenced the results [9]. These systemic mechanisms, although traditionally associated with oral or injectable glucocorticoids, are increasingly recognized as relevant to topical formulations due to percutaneous absorption. Therefore, even minimal systemic exposure from topical corticosteroids may be sufficient to trigger glucose dysregulation in susceptible individuals.

This study has several strengths. It is the first study, to our knowledge, to investigate the association between topical corticosteroid use and the risk of diabetes in an Asian population. It also validates a consistent risk of diabetes development previously observed in studies conducted on Caucasian populations. Furthermore, as this research is based on a large‐scale nationwide dataset from Korea, it holds significant clinical value and provides robust evidence applicable to broader populations. However, this study has certain limitations. There is potential for selection bias, as individuals without health examination records were excluded from the analysis. Thus, another limitation of this study is the exclusion of individuals without health examination records, which may affect the generalizability of the findings. However, South Korea operates a universal national health screening program, and the participation rate among adults aged 19 and older is relatively high—estimated at around 70%–80% in recent years. Therefore, our study population is likely to be broadly representative of the general adult population. Nonetheless, individuals who do not undergo health examinations may differ in health status, access to care, or socioeconomic characteristics, which could introduce some degree of selection or information bias. Furthermore, we included participants with a history of systemic corticosteroid use to reflect real‐world clinical practice, where topical and systemic corticosteroids are frequently prescribed concurrently. Although these individuals may inherently carry a higher risk of diabetes, we addressed this potential confounding by adjusting for systemic corticosteroid use in the multivariable models. Nonetheless, we acknowledge the possibility of residual confounding, and future studies could consider exclusion or stratification approaches to further refine causal inference. The definition of topical corticosteroid use relied on prescription counts, which may not accurately reflect the actual amount used or the area of application among participants. Moreover, as our study relied on administrative prescription data, we could not assess actual patient adherence to prescribed topical corticosteroids. This limitation introduces the potential for exposure misclassification, which may have biased the observed association toward the null. Another limitation of our exposure measurement is the lack of direct data on cumulative dose and treatment duration. Although topical corticosteroid prescriptions may vary in content, they are commonly issued in standardized quantities for short‐term use (e.g., 2–4 weeks) in Korea. Therefore, the number of prescriptions is likely to serve as a reasonable proxy for both exposure frequency and cumulative dose. Nonetheless, future studies with access to detailed dispensing records should consider evaluating defined daily doses (DDD) or treatment duration to better quantify cumulative exposure. Furthermore, the study did not account for the timing of initiation or discontinuation of both topical and systemic corticosteroid use, which may have influenced the observed associations. Despite adjusting for a wide range of confounders, the possibility of residual confounding remains, as unmeasured factors or imperfect measurement of included covariates may still influence the association. We acknowledge that the complex nature of corticosteroid use and metabolic risk makes complete adjustment challenging in observational data. While formal sensitivity analyses such as propensity score matching or exclusion of early events could enhance the robustness of findings, these were not performed in the current study. Future analyses incorporating such designs would further validate the observed associations. Finally, subgroup analyses by age, sex, or comorbidities were not performed in the current study. Given the potential for heterogeneous effects across demographic and clinical subgroups, future research should investigate whether the risk associated with topical corticosteroid use differs by these factors.

## 5. Conclusions

This study confirmed that the risk of developing Type 2 diabetes increases with the use of topical corticosteroids, depending on both usage and frequency. Therefore, before initiating topical corticosteroid treatment, it is important to screen for impaired glucose tolerance and identify high‐risk groups, allowing for the consideration of alternative treatments. If no alternatives are available, regular monitoring during treatment, education on the symptoms of diabetes and steroid‐induced diabetes, as well as efforts to mitigate other risk factors, are essential. To prevent Type 2 diabetes and ensure the safe use of topical corticosteroids, it is necessary to establish guidelines based on more systematic research as soon as possible.

## Author Contributions

Seungwoo Kim and Ji Hui An conceptualized and designed the study and analyzed the data. In Sun Ryou provided clinical expertise. The manuscript was initially prepared by Yongho Jee.

## Funding

This research was conducted with support from the National Research Foundation of Korea with funding from the Ministry of Science and ICT (No. RS‐2023‐00210888).

## Disclosure

All authors have revised and approved the final version of the paper and agree to be accountable for all aspects of their work.

## Ethics Statement

This study was approved by the Institutional Review Board of Yonsei University Health System (IRB No. Y‐2019‐0058). The requirement for informed consent was waived because the study used de‐identified data from the National Health Insurance Service (NHIS) claims database, which is publicly available for research purposes. All data were fully anonymized prior to access.

## Conflicts of Interest

The authors declare no conflicts of interest.

## Supporting Information

Additional supporting information can be found online in the Supporting Information section.

## Supporting information


**Supporting Information** Supporting Table 1. Variables and Definitions Used in the Study. Supporting Table 2. Types of systemic corticosteroids and their primary ingredient codes. Supporting Table 3. Classification of Topical Corticosteroids by Potency and Primary Ingredient Codes. Supporting Table 4. Characteristics According to the Frequency of Topical Corticosteroid use. Supporting Table 5. Characteristics according to the Potency of Topical Corticosteroids.

## Data Availability

The data that support the findings of this study are available from the National Health Insurance Service (NHIS) of Korea. However, these data are not publicly available and are accessible only upon reasonable request and with permission from the NHIS.
