# Supplementary material for: Long‐Term Risk of Type 2 Diabetes Associated With Topical Corticosteroid Use: A Nationwide Population‐Based Cohort Study in Korea
Source: Adv Pharmacol Pharm Sci. 2026 May 28;2026:8843438. doi: 10.1155/adpp/8843438 (PMC13216858; doi:10.1155/adpp/8843438)
Supplement: Supplementary file 1 — Supporting Information Supporting Table 1. Variables and Definitions Used in the Study. Supporting Table 2. Types of systemic corticosteroids and their primary ingredient codes. Supporting Table 3. Classification of Topical Corticosteroids by Potency and Primary Ingredient Codes. Supporting Table 4. Characteristics According to the Frequency of Topical Corticosteroid use. Supporting Table 5. Characteristics according to the Potency of Topical Corticosteroids. [file ADPP-2026-8843438-s001.docx]

Supplementary table 1. Variables and Definitions Used in the Study

|  | Variable | Definition | Reference group |
| --- | --- | --- | --- |
| Independent  Variables | Topical Corticosteroid Use | Prescribed topical corticosteroids two or more times in outpatient care during 2006-2007. | - |
|  | Age | Grouped into: under 30, 30-39, 40-49, 50-59, 60-69, 70 or older. | under 30 |
|  | Gender | Male / Female | Male |
|  | Body Mass Index (BMI) | Body weight (kg) / height (m²) | - |
|  | Smoking Status | Categorized into non-smoker and ever-smoker. | Non-smoker |
|  | Alcohol Consumption | "Consumes alcohol 2-3 times a month or drinks rarely" is considered non-drinker. | Non-drinker |
|  | Atopic Dermatitis | Defined by a primary or secondary diagnosis code L40.x (ICD-6: Atopic Dermatitis) at least once. | - |
| covariates | Income Level | Reclassified into 5 quintiles from the 10 insurance premium levels: Lowest (0-2nd), Below average (3-4th), Average (5-6th), Above average (7-8th), Highest (9-10th). | Average (5-6th) |
|  | Family History of Diabetes | Positive family history of diabetes in health examination records. | - |
|  | Hypertension | Positive history of hypertension in health examination records or past medical history. | - |
|  | Hyperlipidemia | Total cholesterol level ≥ 240 mg/dL. | - |
|  | Fasting Blood Glucose | Classified as under 100 mg/dL or 100-125 mg/dL, excluding individuals with ≥ 126 mg/dL from the study. | under 100 mg/dL |
|  | Use of Systemic Corticosteroids | Prescribed systemic corticosteroids at least once in outpatient care during 2006-2007. | - |
| Outcome Variables | Type 2 Diabetes Mellitus | Meeting one of the following criteria between 2008-2015: (1) Diagnosed with E11.x (ICD-6: Type 2 Diabetes) at least 3 times as a primary or secondary diagnosis, or (2) Prescribed hypoglycemic agents at least once. | - |

Supplementary table 2. Types of systemic corticosteroids and their primary ingredient codes

| Category | Drug Name | Primary Ingredient Code (2008.03) |
| --- | --- | --- |
| Betamethasone | betamethasone 250㎍ | 296900ATB |
|  | betamethasone 500㎍ | 116401ATB |
|  | betamethasone sodium phosphate 4mg | 116502BIJ |
|  | betamethasone sodium phosphate 500㎍ | 116501ATB |
| Budesonide | budesonide 20mg | 119402CAE |
|  | budesonide 3mg | 119406ACH |
|  | budesonide 500㎍ | 119404CSI |
|  | budesonide(micronized) 2.3mg | 119501CTB |
|  | budesonide(micronized) 20mg | 119502CSI |
|  | budesonide(micronized) 40mg | 119505CSI |
| Deflazacort | deflazacort 6mg | 140801ATB |
| Dexamethasone | dexamethasone 4mg | 141904ATB |
|  | dexamethasone 500㎍ | 141901ATB |
|  | dexamethasone 750㎍ | 141903ATB |
|  | dexamethasone palmitate 4mg | 142001BIJ |
|  | dexamethasone sodium phosphate 4.37mg | 142202BIJ |
|  | dexamethasone sodium phosphate 5mg | 142201BIJ |
| Fludrocortisone | fludrocortisone acetate 100㎍ | 160201ATB |
| Fluorometholone | fluorometholone 1mg | 161201COS |
| Fluticasone | fluticasone propionate 15mg | 162202CSI |
|  | fluticasone propionate 15mg | 334600CSI |
|  | fluticasone propionate 20mg | 407100CSI |
|  | fluticasone propionate 30mg | 162204CSI |
|  | fluticasone propionate 30mg | 334700CSI |
|  | fluticasone propionate 40mg | 407200CSI |
|  | fluticasone propionate 6mg | 162207CSI |
|  | fluticasone propionate 6mg | 162205CSI |
|  | fluticasone propionate 6mg | 334500CSI |
|  | fluticasone propionate 8mg | 407300CSI |
| Methylprednisolone | methylprednisolone 16mg | 193301ATB |
|  | methylprednisolone 4mg | 193302ATB |
| Methylprednisolone aceponate | methylprednisolone aceponate 2mg | 193304ATB |
|  | methylprednisolone acetate 200mg | 193501BIJ |
|  | methylprednisolon eacetate 40mg | 193502BIJ |
|  | methylprednisolone sodium succinate 125mg | 193601BIJ |
|  | methylprednisolone sodium succinate 40mg | 193603BIJ |
|  | methylprednisolone sodium succinate 500mg | 193604BIJ |
| Mometasone furoate | mometasonefuroate 50mcg/dose×140dose | 361301CSI |
| Prednisolone | prednisolone 1mg | 217003ASY |
|  | prednisolone 3mg | 217004ASY |
|  | prednisolone 5mg | 217001ATB |
|  | prednisolone sodium succinate 250mg | 217302BIJ |
| Triamcinolone | triamcinolone 1mg | 243201ATB |
|  | triamcinolone 2mg | 243202ATB |
|  | triamcinolone 4mg | 243203ATB |
| Triamcinolone acetonide | triamcinolone acetonide 200mg | 243301BIJ |
|  | triamcinolone acetonide 40mg | 243303BIJ |
|  | triamcinolone acetonide 5.5mg | 243308CSI |
|  | triamcinolone acetonide 50mg | 243305BIJ |

Supplementary Table 3. Classification of Topical Corticosteroids by Potency and Primary Ingredient Codes

| Category | | Drug Name | Primary Ingredient Code (2008.03) |
| --- | --- | --- | --- |
| High strength | Class 1 | clobetasol propionate 466㎍ | 135801CLQ |
|  |  | clobetasol propionate 500㎍ | 339300COM |
|  |  | clobetasol propionate 500㎍ | 135802CCM |
|  |  | clobetasol propionate 500㎍ | 135802CLT |
|  |  | clobetasol propionate 500㎍ | 135802COM |
|  |  | clobetasone butyrate 500㎍ | 135901CCM |
|  |  | clobetasone butyrate 500㎍ | 135901COM |
|  |  | diflorasonedi acetate 500㎍ | 144401CCM |
|  |  | diflucortolone valerate 1mg | 345500CCM |
|  |  | diflucortolone valerate 3mg | 144501COM |
|  | Class 2 | mometasone furoate 1mg | 361302CCM |
|  |  | mometasone furoate 1mg | 361302CLT |
|  |  | mometasone furoate 1mg | 361302COM |
|  |  | fluocinonide 500㎍ | 160801CLQ |
|  |  | halcinonide 1mg | 167801CCM |
|  |  | halcinonide 1mg | 167801COM |
| Mid strength | Class 3 | betamethasone dipropionate 0.643mg | 490500COM |
|  |  | betamethasone dipropionate 1mg | 345000CCM |
|  |  | betamethasone dipropionate 640㎍ | 344900CCM |
|  |  | betamethasone dipropionate 640㎍ | 346400CCM |
|  |  | betamethasone dipropionate 640㎍ | 346400COM |
|  |  | amcinonide 1mg | 106401CCM |
|  | Class 4 | methylprednisolone aceponate 1mg | 193401CCM |
|  |  | methylprednisolone aceponate 1mg | 193401CLT |
|  |  | methylprednisolone aceponate 1mg | 193401COM |
|  |  | budesonide 10mg | 119405CLQ |
|  |  | budesonide 1mg | 119401CLQ |
|  |  | budesonide 250㎍ | 119403CCM |
|  |  | fluocinolone acetonide 250㎍ | 160701CCM |
|  |  | fluocinolone acetonide 0.25mg | 475900CLQ |
|  |  | triamcinolone acetonide 1mg | 243302COM |
|  |  | triamcinolone acetonide 1mg | 243302CPA |
|  |  | triamcinolone acetonide 1mg | 342800CCM |
|  |  | triamcinolone acetonide 1mg | 342800COM |
|  |  | triamcinolone acetonide 1mg | 243304CCM |
|  |  | triamcinolone acetonide 1mg | 243304COM |
|  |  | triamcinolone acetonide 6.6mg | 243309CLQ |
|  | Class 5 | prednicarbate 1mg | 216902CCM |
|  |  | prednicarbate 2.5mg | 216901CLQ |
|  |  | prednicarbate 2.5mg | 216901CLT |
|  |  | prednicarbate 2.5mg | 216901CCM |
|  |  | prednicarbate 2.5mg | 216901COM |
|  |  | betamethasone valerate 1.105mg | 116601CLQ |
|  |  | fluticasone propionate 0.5mg | 162201CCM |
|  |  | fluticasone propionate 2mg | 162203CLQ |
| Mild strength | Class 6 | alclometasone dipropionate 0.5mg | 103401CCM |
|  |  | alclometasone dipropionate 1mg | 103402CCM |
|  |  | desonide 500㎍ | 141501CCM |
|  |  | desonide 500㎍ | 141501CLT |
|  | Class 7 | prednisolone 2.5mg | 217002CCM |
|  |  | prednisolone 2.5mg | 217002CLT |
|  |  | prednisolone valerate 3mg | 217501CCM |
|  |  | prednisolone valerate 3mg | 217501CLT |
|  |  | prednisolone valerate 3mg | 217501COM |
|  |  | dexamethasone propionate 1mg | 142101CCM |

Supplementary table 4. Characteristics According to the Frequency of Topical Corticosteroid use

|  | **TCS Frequency per 2 years (Mean)^*^** | | | |
| --- | --- | --- | --- | --- |
|  | **0** | **1** | **2-4 (2.5)** | **5≤ (6.9)** |
| n (%) | 145,149 (78.0) | 25,964 (14.0) | 13,105 (7.0) | 1,839 (1.0) |
| Total person year | 1,247,343 | 223,059 | 111,388 | 15,261 |
| Mean person year | 8.59 | 8.59 | 8.50 | 8.30 |
| Sex | N (%) | N (%) | N (%) | N (%) |
| Male | 82,834 (57.1) | 12,064 (46.5) | 5,690 (43.4) | 818 (44.5) |
| Female | 62,315 (42.9) | 13,900 (53.6) | 7,415 (56.6) | 1,021 (55.5) |
| Age(year) |  |  |  |  |
| Mean (SD) | 42.1(14.0) | 42.7(14.2) | 44.2(14.4) | 47.2 (15.1) |
| <30 | 32,369 (22.3) | 5,619 (21.6) | 2,554 (19.5) | 277 (15.1) |
| 30 - 39 | 36,024 (24.8) | 6,076 (23.4) | 2,750  21.0 | 323  17.6 |
| 40 - 49 | 36,761 (25.3) | 6,605 (25.4) | 3,356 (25.6) | 471 (25.6) |
| 50 - 59 | 21,947 (15.1) | 4,186 (16.1) | 2,362 (18.0) | 357 (19.4) |
| 60 - 69 | 12,119 (8.3) | 2,374 (9.1) | 1,423 (10.9) | 252 (13.7) |
| 70 | 5,929 (4.1) | 1,104 (4.3) | 660 (5.0) | 159 (8.6) |
| BMI (kg/m^2^) |  |  |  |  |
| Mean (SD) | 23.5(3.3) | 23.4 (3.2) | 23.5(3.2) | 23.6(3.3) |
| <18.5 | 6,939 (4.8) | 1,330 (5.1) | 664 (5.1) | 85 (4.6) |
| 18.5-25 | 93,413 (64.4) | 16,820 (64.8) | 8,360 (63.8) | 1,169 (63.6) |
| 25-30 | 39,833 (27.4) | 8,360 (27.1) | 3,671 (28.0) | 514 (28.0) |
| >30 | 4,938 (3.4) | 1,169 (3.0) | 410 (3.1) | 70 (3.8) |
| Smoking |  |  |  |  |
| Ever-smoker | 61,614 (42.9) | 8,937 (34.7) | 4,253 (32.8) | 608 (33.6) |
| Nonsmoker | 82,070 (57.1) | 16,785 (65.3) | 8,724 (67.2) | 1,202 (66.4) |
| Alcohol |  |  |  |  |
| 1–2 times/week | 40,506 (28.2) | 6,353 (24.7) | 3,061 (23.6) | 400 (22.1) |
| <2–3 times/month | 103,121 (71.8) | 19,364 (75.3) | 9,910 (76.4) | 1,411 (77.9) |
| FBS (mg/dl) (SD) | 93.7(17.0) | 93.0(15.5) | 93.2(15.7) | 93.9(16.0) |
| <100 | 107,953 (74.4) | 19,780 (76.2) | 9,867 (75.3) | 1,360 (74.0) |
| 100-125 | 33,347 (23.0) | 5,605 (21.6) | 2,935 (22.4) | 423 (23.0) |
| Psoriasis | 220 (0.2) | 316 (1.2) | 335 (2.6) | 160 (8.7) |
| Hypertension | 13,910 (9.6) | 2,726 (10.5) | 1,547 (11.8) | 274 (14.9) |
| Hyperlipidemia | 17,709 (12.2) | 3,285 (12.7) | 1,749 (13.3) | 275 (15.0) |
| DM family history | 24,506 (16.9) | 4,358 (16.8) | 2,060 (15.7) | 307 (16.7) |
| Systemic CS | 16,463 (11.3) | 5,701 (22.0) | 3,784 (28.9) | 779 (42.4) |
| Income status |  |  |  |  |
| Lowest | 23,525 (16.6) | 4,221 (43.3) | 2,218 (17.4) | 317 (18.2) |
| Below average | 25,102 (17.7) | 4,224 (16.7) | 2,129 (16.7) | 298 (17.1) |
| Average | 27,964 (19.8) | 4,747 (18.7) | 2,336 (18.3) | 305 (17.5) |
| Above average | 32,688 (23.1) | 5,924 (23.4) | 2,947 (23.1) | 397 (22.8) |
| Highest | 32,300 (22.8) | 6,234 (24.6) | 3,120 (24.5) | 422 (24.3) |
| Potency |  |  |  |  |
| Mild |  | 5,094 (19.6) | 2,485 (19.0) | 308 (16.7) |
| Mid |  | 9,636 (37.1) | 4,846 (37.0) | 655 (35.6) |
| High |  | 11,234 (43.3) | 5,774 (44.1) | 876 (47.6) |
| T2D incidence  during F/U | **10,304 (7.1)** | **2,014 (7.8)** | **1,138 (8.7)** | **223 (12.1)** |

Abbreviations: BMI, body mass index (kg/m²); DM: Diabetes mellitus; FBS, fasting blood sugar (mg/dL; CS, corticosteroid.

Supplementary table 5. Characteristics according to the Potency of Topical Corticosteroids

|  | **Non-use** | **Use (by TCS potency)** | | |
| --- | --- | --- | --- | --- |
|  |  | **Mild** | **Mid** | **High** |
| N (%) | 171,113 (92.0) | 2,793 (1.5) | 5,501 (3.0) | 6,550 (3.5) |
| Total follow-up time, PY | 1,470,402 | 23,791 | 46,653 | 56,206 |
| Mean follow-up time, years | 8.59 | 8.52 | 8.48 | 8.45 |
| TCS Frequency (SD) |  | 2.97(1.8) | 2.97(1.8) | 3.01(2.1) |
|  | N % | N % | N % | N % |
| Sex |  |  |  |  |
| Male | 94,898 (55.5) | 1,059 (37.9) | 2,482 (45.1) | 2,967 (45.3) |
| Female | 76,215 (44.5) | 1,734 (62.1) | 3,019 (54.9) | 3,683 (54.7) |
| Age(year) |  |  |  |  |
| Mean (SD) | 42.2(14.0) | 44.4(14.8) | 44.8(14.4) | 45.3(14.4) |
| <30 | 37,988 (22.2) | 675 (24.2) | 1,006 (18.3) | 1,150 (17.6) |
| 30 - 39 | 42,100 (24.6) | 627 (22.4) | 1,154 (21.0) | 1,292 (19.7) |
| 40 - 49 | 43,366 (25.3) | 673 (24.1) | 1,370 (24.9) | 1,784 (27.2) |
| 50 - 59 | 26,133 (15.3) | 415 (14.9) | 1,053 (19.1) | 1,251 (19.1) |
| 60 - 69 | 14,493 (8.5) | 261 (9.3) | 629 (11.4) | 785 (12.0) |
| ≥70 | 7,033 (4.1) | 142 (5.1) | 289 (5.3) | 388 (5.9) |
| BMI (kg/m^2^) |  |  |  |  |
| Mean (SD) | 23.5(3.3) | 23.2(3.3) | 23.6(3.3) | 23.6(3.1) |
| <18.5 | 8,269 (4.8) | 197 (7.1) | 262 (4.8) | 290 (4.4) |
| 18.5-25 | 110,233 (64.4) | 1,801 (64.5) | 3,445 (62.6) | 4,283 (64.4) |
| 25-30 | 46,871 (27.4) | 719 (25.7) | 1,593 (29.0) | 1,873 (28.2) |
| >30 | 5,713 (3.3) | 76 (2.7) | 201 (3.7) | 203 (3.1) |
| Smoking |  |  |  |  |
| Ever-smoker | 70,551 (41.6) | 827 (29.9) | 1,823 (33.5) | 2,211 (33.6) |
| Nonsmoker | 98,855 (58.4) | 1,938 (70.1) | 3,616 (66.5) | 4,372 (66.4) |
| Alcohol |  |  |  |  |
| More than 1~2 times/Week | 46,859 (27.7) | 610 (22.1) | 1,308 (24.1) | 1,543 (23.4) |
| Less than 2~3 times/Month | 122,485 (72.3) | 2,154 (77.9) | 4,123 (75.9) | 5,044 (76.6) |
| FBS (mg/dl) (SD) | 93.6 (16.8) | 92.6 (16.6) | 93.6 (15.9) | 93.3 (15.2) |
| <100 | 127,733 (74.6) | 2,141 (76.7) | 4,095 (74.4) | 4,991 (76.2) |
| 100-125 | 38,952 (22.8) | 588 (21.1) | 1,258 (22.9) | 1,512 (23.1) |
| Psoriasis | 536 (0.31) | 65 (2.33) | 159 (2.89) | 271 (4.14) |
| Hypertension | 16,636 (9.7) | 295 (10.6) | 700 (12.7) | 826 (12.4) |
| Hyperlipidemia | 20,994 (12.3) | 333 (11.9) | 775 (14.1) | 916 (13.8) |
| DM family history | 28,864 (16.9) | 508 (18.2) | 849 (15.4) | 1,010 (15.2) |
| Systemic CSs | 22,164 (13.0) | 878 (31.4) | 1,618 (29.4) | 2,067 (31.6) |
| Income status |  |  |  |  |
| Lowest | 15,305 (9.2) | 260 (9.5) | 486 (9.1) | 618 (9.6) |
| Below average | 12,441 (7.5) | 217 (8.0) | 452 (8.5) | 502 (7.8) |
| Average | 12,519 (7.5) | 207 (7.6) | 370 (6.9) | 455 (7.1) |
| Above average | 16,807 (10.1) | 267 (9.8) | 510 (9.6) | 618 (9.6) |
| Highest | 16,976  (10.2) | 264  (9.7) | 479  (9.0) | 588 (9.2) |
| **T2D incidence during F/U** | **12,318 (7.2)** | **226 (8.1)** | **535 (9.7)** | **600 (9.0)** |

Abbreviations: SD, standard deviation; BMI, body mass index (kg/m²); FBS, fasting blood sugar (mg/dL); T2D, type 2 diabetes; F/U, follow-up; CS, corticosteroid; TCS, topical corticosteroids..
